# Predictors of Return to Work Following Major Trauma in New Zealand

**DOI:** 10.1111/1742-6723.70382

**Published:** 2026-10-29

**Authors:** Duncan Finlayson, Sarah Logan, Daniel Jemberie, Nikita Quinn, Laura R. Joyce, Christopher Wakeman, Andrew McCombie

**Affiliations:** ^1^ Emergency Department Te Whatu Ora Waitaha Canterbury Christchurch New Zealand; ^2^ Department of Surgery Te Whatu Ora Waitaha Canterbury Christchurch New Zealand; ^3^ Department of Surgery and Critical Care University of Otago Christchurch New Zealand

**Keywords:** anxiety, depression, PTSD, return to work, trauma

## Abstract

**Objective:**

This study aimed to identify physical and psychological predictors of return to work (RTW) in a cohort of major trauma patients in New Zealand.

**Methods:**

A retrospective cohort study was conducted at Christchurch Hospital involving patients identified from the New Zealand Major Trauma Registry. Full‐time workers (*n* = 130) who sustained major trauma between 2016 and 2020 completed self‐reported questionnaires assessing post‐injury work capacity, PTSD (PCL‐5), anxiety and depression (HADS) and perceived social support (MSPSS). Univariable and multivariable analyses were performed to determine predictors of decreased RTW following injury.

**Results:**

Older age, longer hospital length of stay (LOS) and ICU admission were all significantly associated with decreased RTW in the univariable analysis. Probable anxiety and depression were also significantly associated with decreased RTW, while meeting PTSD thresholds did not yield a significant association. In the final multivariable analysis, older age (OR 1.08, *p* < 0.01), longer LOS (OR 1.04, *p* = 0.048) and those with probable depression (OR 5.29, *p* = 0.01) were significantly associated with decreased RTW.

**Conclusions:**

Probable depression is a strong, independent psychological predictor of reduced RTW following major trauma. These findings highlight the need for integrated post‐trauma care models that incorporate routine psychological screening and early intervention to address both the physical and psychological needs of patients suffering from major trauma.

**Key Findings**

- Physical and psychological predictors of return to work were identified following major trauma in a New Zealand cohort.
- Probable depression emerged as the most potent predictor of decreased work in the multivariable analysis.
- This study highlights the importance of integrated post‐trauma care that tends to the physical and psychological needs of injured patients.

## 1 Introduction

Major trauma is a leading cause of injury‐related disability and death worldwide [1]. Major trauma can cause a significant reduction in health‐related quality of life due to both physical and psychological morbidities [2]. The long‐term effects of major trauma on physical health include chronic pain and physical disabilities, resulting in a diminished capacity to perform basic activities of daily living [3]. While the effects of major trauma on physical health are well established, the psychological impacts remain comparatively less understood.

Psychological illnesses, such as post‐traumatic stress disorder (PTSD), major depressive disorder and anxiety disorders, are common after traumatic injuries [4, 5, 6, 7]. Several studies have identified that psychological sequelae are often under‐diagnosed following major trauma [4, 8, 9, 10]. Failing to identify and treat psychological illness following major trauma may lead to a reduced quality of life for patients and impose considerable costs on individuals and society [2].

Rehabilitation following major trauma is crucial for restoring function, improving quality of life and supporting the physical and emotional recovery of individuals. Rehabilitation is a costly and time‐consuming process for both individuals and society, making it essential to maximise efficiency in order to shorten the rehabilitation period for patients [11]. The primary goal of rehabilitation is to return people to their premorbid functioning—which in working individuals, includes returning to work (RTW). RTW is vital for an individual's physical and psychological well‐being [12]. Aotearoa New Zealand is unique in that a Crown entity insurance scheme, the Accident Compensation Corporation (ACC), provides compensation and funded healthcare to those with personal injuries. ACC supports patients with income compensation during a period of graduated RTW. The aim of this scheme is to support patients RTW early after injury [13].

The physical predictors of RTW following major trauma have been widely studied, with research identifying several key determinants. The severity of physical injury has been correlated with extended delays in RTW [14]. Admission to intensive care units, the necessity for mechanical ventilation and length of hospital stay have all been associated with delays in RTW [15, 16]. The mechanism of trauma has also been implicated, with motor vehicle accidents being a positive predictor of delayed RTW [17].

While the physical factors impacting RTW following major trauma have been extensively investigated, there is a paucity of data on the psychological factors impacting RTW. Furthermore, more analysis into factors predicting RTW in a New Zealand specific context, such as the impact of ACC, is required. As such, this study reviews factors predicting the ability to RTW in a cohort of New Zealand patients—particularly focused on psychological factors.

## 2 Methods

### 2.1 Study Design

This was a single‐centre retrospective cohort study exploring the factors associated with RTW following traumatic injury in a New Zealand cohort.

### 2.2 Setting

This study was set in Christchurch Hospital, a tertiary hospital and the only major trauma centre in the South Island of New Zealand [18]. It serves an estimated population of 600,000 people but also takes transfers from other areas in New Zealand for spinal and burn injuries. The emergency department sees over 130,000 presentations and over 400 major trauma cases per year.

### 2.3 Participants

Participants were identified from the New Zealand Major Trauma Registry (NZMTR) between May 2016 and March 2020 [19]. Participants aged 16 or older were included if admitted to hospital as an inpatient within 7days of traumatic injury, had an Injury Severity Score (ISS) of greater than 12, and sustained the trauma at least 6 months prior to recruitment. Participants were excluded if their injury was non‐traumatic, an attempted suicide, secondary to poisoning or exertional injury without external force, or attributable to pathological or pre‐existing conditions. Patients with severe traumatic brain injury (Glasgow coma scale < 8) were excluded because making diagnosing PTSD by self‐reported questionnaires in this cohort can be challenging. Victims of the Christchurch Mosque attacks on 15th March 2019 were also excluded.

### 2.4 Variables and Data Sources

Eligible participants were mailed an information sheet and consent form in March 2020. All people were contacted one extra time (twice total) at the stage of obtaining consent. Those who returned their consent forms were then mailed the study questionnaires. If participants failed to return their questionnaires, they were followed up with two phone calls. If there was no response after the second follow‐up phone call, no further attempt to make contact was made.

Participants who consented were mailed a six‐point questionnaire relating to their ability to work before and after their traumatic injury (Supporting Information 1), so was partly relying on their retrospective recall. The questionnaire was tested for face validity on three lay people before being used in the study and contained two questions asking about current work status and capacity. Questions were formulated to assess the participant's post‐injury work capacity, specifically in terms of hours worked and productivity levels. Participants were categorised into those who could not work at all following injury, and those who continued to work but with decreased hours or reported decreased productivity, compared to their pre‐injury work status. Decreased productivity refers to those who returned to work the same number of hours but reported completing less work within this time. For the purposes of the first univariable analysis, those classified as having ‘decreased work’ were a combination of those who could not work at all and those who continued to work but with decreased hours and reported decreased productivity.

PTSD data was collected using a questionnaire based on the PTSD checklist for DSM‐5 (PCL‐5) [20, 21]. This is a 20‐item self‐report measure that assesses DSM‐5 symptoms on a four‐point scale. A PCL‐5 cut‐off of greater than 30 for probable PTSD was used. This was measured at follow up only.

Anxiety and depression were measured using the Hospital Anxiety and Depression Scale (HADS) [22]. This is a 14‐item self‐report questionnaire used for screening patients in a hospital (outpatient and inpatient) context for anxiety and depression. Symptoms are rated on a four‐point Likert scale. It omits questions related to symptoms that may be caused by physical illness (e.g., dizziness) and concentrates on psychological aspects of anxiety and depression. A cut‐off of eight or greater was used to indicate probable anxiety and depression. These were measured at follow up only (i.e., not before or immediately after the injury).

Participants were asked two questions relating to pre‐existing mental illness and treatment to account for any mental illness which may have predated the trauma. These questions were asked at follow up and therefore required retrospective answers.

Those meeting the cut‐offs as described previously were classified as having “probable” PTSD, anxiety and depression.

Social support was assessed using the Multidimensional Scale of Perceived Social Support (MSPSS) [23]. This 12‐item scale assesses perceptions of social support. Statements indicative of perceived support are rated on a seven‐point Likert scale. Questions determined the levels of support from significant others, family and friend subgroups. Each subgroup had four questions relating to it. Mean scores were calculated for each subgroup. Higher scores indicate greater levels of perceived social support [23]. This was measured at the time of follow up only.

Demographics including age, gender, ethnicity, injury type and severity, mechanism of injury, level of consciousness at time of injury, operations and procedures and LOS were obtained from the NZMTR as well as clinical records (both collected at the time of injury and hospitalisation). The acute use of alcohol directly prior to trauma was determined from clinical records on admission.

### 2.5 Study Size

The expected sample size was 650 [7].

### 2.6 Statistical Analysis

SPSS version 29 was used for the analyses. Responders were compared to non‐responders and the statistical tests were footnoted in the relevant Supporting Information accordingly. Univariable analyses were performed for overall decreased work and its component parts—that being a total inability to work following injury, RTW but with decreased hours, and RTW but with reported decreased productivity. For univariable analyses two‐sided Pearson Chi squares with continuity corrections were used for 2*2 crosstabulations, two‐sided Fisher–Freeman–Halton exact tests were used for > 2*2 crosstabulations, and two‐sided Mann Whitney U tests were used for comparing continuous data reported with medians. A final backwards step multivariable model was performed to determine the variables associated with decreased work following the injury. The criteria used were PIN 0.05, POUT 0.05, iterate 20 and cut 0.5, with age and sex being entered unconditionally. Two composite variables were considered throughout: self‐reported PTSD, anxiety, or depression before the injury (i.e., at least one of the three) and scores at follow up after the injury (i.e., crossing the threshold for at least one of the three).

## 3 Results

A total of 1286 major trauma patients admitted to Christchurch Hospital between May 2016 and March 2020 were screened for this study. Of these, 450 did not meet inclusion criteria. Among the remaining 836 eligible patients, 599 declined to participate. Ultimately, 203 participants (24.3% response rate) completed and returned the questionnaire (Tables 1 and S1). This cohort was predominantly male and Caucasian. The most common mechanism of trauma was vehicle‐related.

**TABLE 1:** Demographics of participants.

Variable	Full time (*n* = 130)	Part time (*n* = 28)	Other (*n* = 45)
Sex			
Female	32 (24.6%)	12 (42.9%)	20 (44.4%)
Male	98 (75.4%)	16 (57.1%)	25 (55.6%)
Ethnicity			
Caucasian	117 (90%)	28 (100%)	43 (95.6%)
Māori	5 (3.8%)	0 (0%)	2 (4.4%)
Other	8 (6.2%)	0 (0%)	0 (0%)
Median (interquartile range) Age	52 (43.75–59)	59.5 (54–71)	73 (54–77)
Years between injury and response	3.03 (2.55–3.61)	3.06 (2.47–3.84)	2.82 (2.23–3.58)
Median (interquartile range) ISS Score	16 (13–20)	16 (13–22)	16 (14–20.5)
< 16	47 (36.2%)	11 (39.3%)	17 (37.8%)
≥ 16	83 (63.8%)	17 (60.7%)	28 (62.2%)
Mechanism			
Vehicle‐related	68 (52.3%)	15 (53.6%)	24 (53.3%)
Sport‐related	42 (32.3%)	10 (35.7%)	9 (20%)
Fall	15 (11.5%)	2 (7.1%)	12 (26.7%)
Work‐place related	7 (5.4%)	1 (3.6%)	0 (0%)
Assault	1 (0.8%)	0 (0%)	0 (0%)
Unknown	0 (0%)	1 (3.6%)	0 (0%)
Alcohol Involved	7 (5.4%)	1 (3.6%)	2 (4.4%)
ICU Admission	33 (25.4%)	9 (32.1%)	5 (11.1%)
Hospital LOS (days)	7 (3–13)	9 (4.25–16)	7 (3.5–11.5)
Probable PTSD at follow up	27 (20.8%)	5 (17.9%)	5 (11.1%)
Probable anxiety at follow up	49 (37.7%)	9 (33.3%)	12 (26.7%)
Probable depression at follow up	33 (25.4%)	9 (32.1%)	8 (18.6%)
Probable PTSD, anxiety, or depression at follow up	56 (43.1%)	13 (46.4%)	14 (31.8%)
Pre‐injury PTSD	2 (1.5%)	0 (0%)	4 (8.9%)
Pre‐injury anxiety	10 (7.7%)	5 (17.9%)	4 (8.9%)
Pre‐injury depression	30 (23.1%)	5 (17.9%)	11 (24.4%)
Pre‐injury PTSD, anxiety, or depression	33 (25.4%)	8 (28.6%)	13 (28.9%)
GCS < 15 in ED	17 (13.1%)	4 (14.3%)	9 (20.0%)

*Note:* ‘Full time’ refers to those who were working full time prior to their injury. ‘Part time’ refers to those who were working part time prior to their injury. ‘Other’ refers to those who were not working prior to their injury—and includes pensioners, students, and those not working or studying at all.

Abbreviations: ED, emergency department; GCS, Glasgow coma scale; ICU, intensive care unit; ISS, injury severity score; LOS, length of hospital stay; PTSD, post‐traumatic stress disorder.

Our analysis aimed to identify factors associated with decreased RTW in full‐time workers, compared to pre‐injury work status. Seventy‐three participants were not full‐time workers before their injury, so were excluded for the purposes of our analysis. This left 130 full‐time workers included for analysis (Figure S1).

Results of the first univariable analysis are displayed in Table 2. This analysis identified factors associated with decreased work following injury in full‐time workers. For this analysis, ‘decreased work’ refers to those who could not work at all, or continued work but with decreased hours or reported decreased productivity. Two participants out of 130 gave incomplete answers in the questionnaire so were excluded from this analysis. Older age, LOS, lower family social support and ICU admission were all significantly associated with a decrease in work following injury. Participants with probable anxiety and depression were more likely to have decreased work following injury, but those with probable PTSD were not. When the three mental illnesses were combined, these were associated with decreased work. Participants were screened for pre‐injury mental illness, meaning those meeting thresholds for probable mental illness were assumed to have developed this illness as a direct result of their major trauma. Work‐place related injuries were significantly less likely to result in decreased work. Vehicle‐related injuries had a positive trend towards decreased work, but the result was not statistically significant. No other mechanisms were associated with a significant difference in work status post‐injury.

**TABLE 2:** Univariable (unadjusted) association between explanatory variables and work status of full‐time workers following injury.

Variable	Decreased work (*n* = 55)	No decreased work (*n* = 73)	Test	*p*
Sex			P	
Male	38 (39.6%)	58 (60.4%)		0.26
Female	17 (53.1%)	15 (46.9%)		
Ethnicity			F	
Caucasian	48 (41.7%)	67 (58.3%)		0.49
Māori	2 (40%)	3 (60%)		
Other	5 (62.5%)	3 (37.5%)		
Median age	55 (IQR: 49–60)	49 (IQR: 39–58)	M	0.008
Median time between injury and response	3.19 (2.82–3.70)	2.91 (2.40–3.53)	M	0.056
ISS score			P	
< 16	18 (38.3%)	29 (61.7%)		0.53
≥ 16	37 (45.7%)	44 (54.3%)		
Median LOS	8 (IQR: 5–18)	5 (IQR: 2–10)	M	0.001
Mechanism			P	
Vehicle‐Related	34 (50.7%)	33 (49.3%)		0.092
Not vehicle‐related	21 (34.4%)	40 (65.6%)		
Sport‐related	14 (33.3%)	28 (66.7%)		0.18
Not sport‐related	41 (47.7%)	45 (52.3%)		
Fall	7 (50%)	7 (50%)		0.78
Not fall	48 (42.1%)	66 (57.9%)		
Work‐place related	0 (0%)	7 (100%)		0.049
Not workplace related	55 (45.5%)	66 (54.5%)		
Assault	1 (100%)	0 (0%)		0.89
Not assault	54 (42.5%)	73 (57.5%)		
Alcohol involved			P	
Yes	2 (33.3%)	4 (66.7%)		0.95
No	53 (43.4%)	69 (56.6%)		
ICU admission			P	
Yes	21 (65.6%)	11 (34.4%)		0.005
No	34 (35.4%)	62 (64.6%)		
Admitting specialty			F	
General Surgery	7 (31.8%)	15 (68.2%)		0.08
Cardiothoracics	5 (22.7%)	17 (77.3%)		
Neurosurgery	2 (40%)	3 (60%)		
Orthopaedics – including spine	39 (51.3%)	37 (48.7%)		
All others and unknown	2 (66.7%)	1 (33.3%)		
GCS in ED			P	
15	47 (41.6%)	66 (58.4%)		0.558
< 15	8 (53.3%)	7 (46.7%)		
Probable PTSD at follow up			P	
Above Threshold	13 (52%)	12 (48%)		0.43
Below Threshold	42 (40.8%)	61 (59.2%)		
Probable anxiety at follow up			P	
Above Threshold	29 (60.4%)	19 (39.6%)		0.004
Below Threshold	26 (32.5%)	54 (67.5%)		
Probable depression at follow up			P	
Above Threshold	22 (68.8%)	10 (31.3%)		0.001
Below Threshold	33 (34.4%)	63 (65.6%)		
Probable PTSD, anxiety or depression at follow up				
At least one above threshold	32 (58.2%)	23 (41.8%)	P	0.005
None above threshold	23 (31.5%)	50 (68.5%)		
Pre‐injury PTSD				
Present	2 (100%)	0 (0%)	P	0.36
Absent	53 (42.1%)	73 (57.9%)		
Pre‐injury anxiety				
Present	7 (70%)	3 (30%)	P	0.14
Absent	48 (40.7%)	70 (59.3%)		
Pre‐injury depression				
Present	18 (60%)	12 (40%)	P	0.052
Absent	37 (37.8%)	61 (62.2%)		
Pre‐injury PTSD, depression, or anxiety diagnosis				
At least one Present	19 (57.6%)	14 (42.4%)	P	0.08
None present	36 (37.9%)	59 (62.1%)		
Social support			M	
Significant other	6.25 (5–7)	6.75 (5.25–7)		0.30
Family	5.5 (3.75–6.75)	6.25 (5.25–7)		0.045
Friends	5.5 (4.75–6)	5.75 (6.63–6.5)		0.58

*Note:* ‘Decreased work’ refers to those who could not work at all, or continued work but with decreased hours or reported decreased productivity. Mean scores were calculated for each subgroup (significant others, family, friends). A higher mean score indicates higher levels of perceived support.

Abbreviations: F, Fisher–Freeman Halton Exact test (2 sided); M, Mann Whitney U test (2 sided); P, Pearson Chi square with continuity correction (2‐sided).

Results of univariable subgroup analysis are displayed in Tables 3 and 4. 121 of the 128 participants included in the first univariable analysis answered the self‐report questions about their ability to work, productivity and work hours following injury—seven participants gave incomplete answers, so they were excluded from this subgroup analysis.

**TABLE 3:** Univariable (unadjusted) association between discrete explanatory variables and degrees of work following injury.

Variable	*N* total	Test	Cannot work at all		Decreased hours		Decreased productivity	
			*n* (%)	*p*	*n* (%)	*p*	*n* (%)	*p*
Sex		P						
Male	91		12 (13.2%)	0.86	23 (25.3%)	0.53	21 (23.1%)	0.22
Female	30		5 (16.7%)		10 (33.3%)		11 (36.7%)	
Ethnicity		F						
Caucasian	108		14 (13%)	0.47	27 (25%)	0.15	29 (26.9%)	0.40
Māori	5		1 (20%)		3 (60%)		0 (0%)	
Other	8		2 (25%)		3 (37.5%)		3 (37.5%)	
Mechanism		P						
Vehicle‐related								
Yes	62		14 (22.6%)	0.012	20 (32.3%)	0.290	20 (32.3%)	0.201
No	59		3 (5.1%)		13 (22%)		12 (20.3%)	
Sport‐related								
Yes	40		1 (2.5%)	0.02	8 (20%)	0.296	8 (20%)	0.36
No	81		16 (19.8%)		25 (30.9%)		24 (29.6%)	
Fall								
Yes	14		2 (14.3%)	1.00	5 (35.7%)	0.66	3 (21.4%)	0.90
No	107		15 (14%)		28 (26.2%)		29 (27.1%)	
Work‐place related								
Yes	7		0 (0%)	0.59	0 (0%)	0.22	2 (28.6%)	1.00
No	114		17 (14.9%)		33 (28.9%)		30 (26.3%)	
ISS score		P						
< 16	45		2 (4.4%)	0.039	7 (15.6%)	0.044	13 (28.9%)	0.80
≥ 16	76		15 (19.7%)		26 (34.2%)		19 (25.0%)	
Alcohol involved		P						
Yes	6		1 (16.7%)	1.00	3 (50%)	0.42	1 (16.7%)	0.93
No	115		16 (13.9%)		30 (26.1%)		31 (27%)	
ICU admission		P						
Yes	30		11 (36.7%)	< 0.001	17 (56.7%)	< 0.001	9 (30%)	0.79
No								
Admitting specialty		F						
General surgery	20		2 (10%)	0.38	3 (15%)	0.04	4 (20%)	0.91
Cardiothoracics	22		1 (4.5%)		2 (9.1%)		5 (22.7%)	
Neurosurgery	5		0 (0%)		2 (40%)		1 (20%)	
Orthopaedics – including spine	71		14 (19.7%)		26 (26.6%)		21 (29.6%)	
All others and unknown	3		0 (0%)		0 (0%)		1 (33.3%)	
GCS < 15 in ED		P						
15	107		12 (11.2%)	0.038	27 (25.2%)	0.28	30 (28%)	0.44
< 15	14		5 (35.7%)		6 (42.9%)		2 (14.3%)	
Probable PTSD at follow up		P						
Above threshold	20		5 (25%)	0.23	7 (35%)	0.57	9 (45%)	0.075
Below threshold	101		12 (11.9%)		26 (25.7%)		23 (22.8%)	
Probable anxiety at follow up		P						
Above threshold	43		12 (27.9%)	0.003	22 (51.2%)	< 0.001	18 (41.9%)	0.008
Below threshold	78		5 (6.4%)		11 (14.1%)		14 (17.9%)	
Probable depression at follow up		P						
Above threshold	28		9 (32.1%)	0.005	16 (57.1%)	< 0.001	8 (28.6%)	0.96
Below threshold	93		8 (8.6%)		17 (18.3%)		24 (25.8%)	
Probable PTSD, depression, or anxiety at follow up		P						
At least one above threshold	50		13 (26%)	0.004	24 (48%)	< 0.001	20 (40%)	0.009
None above threshold	71		4 (5.6%)		9 (12.7%)		12 (16.9%)	
Pre‐injury PTSD		P						
Present	2		1 (50%)	0.65	2 (100%)	0.13	1 (50%)	1
Absent	119		16 (13.4%)		31 (26.1%)		31 (26.1%)	
Pre‐injury anxiety		P						
Present	10		4 (40%)	0.047	5 (50%)	0.19	3 (30%)	1
Absent	111		13 (11.7%)		28 (25.2%)		29 (26.1%)	
Pre‐injury depression		P						
Present	29		8 (27.6%)	0.04	12 (41.4%)	0.09	7 (24.1%)	0.94
Absent	92		9 (9.8%)		21 (22.8%)		25 (27.2%)	
Pre‐injury PTSD, depression, or anxiety		P						
Present	32		9 (28.1%)	0.02	13 (40.6%)	0.08	8 (25%)	1
Absent	89		8 (9%)		20 (22.5%)		24 (27%)	

*Note: ‘*Cannot work at all’ are those who did not return to work following injury. ‘Decreased hours’ are those who returned to work but worked less hours in a week following injury. ‘Decreased productivity’ are those who returned to work for the same number of hours per week but reported achieving less at work compared to before the injury.

Abbreviations: F, Fisher–Freeman Halton Exact test (sided); P, Pearson Chi square with continuity correction (2‐sided).

**TABLE 4:** Univariable (unadjusted) association between continuous explanatory variables and degrees of work following injury.

Variable	Cannot work at all			Decreased hours			Decreased productivity		
	Yes	No	*p*	Yes	No	*p*	Yes	No	*p*
Median age (IQR)	52.0 (44.5–60.0)	51.0 (42.0–59.0)	0.46	51.0 (46.0–59.0)	51.0 (39.3–59.0)	0.49	54.5 (39.0–62.5)	51.0 (44.0–59.0)	0.64
Median time between injury and response (IQR)	3.01 (2.54–3.47)	3.02 (2.52–3.61)	0.72	3.01 (2.55–3.57)	3.02 (2.49–3.63)	0.83	3.03 (2.56–3.77)	3.01 (2.51–3.56)	0.49
Median LOS (IQR)	16.0 (7.0–32.5)	6.0 (3.0–11.0)	0.002	12.0 (7.0–20.5)	5.0 (3.0–9.8)	< 0.001	7.0 (3.3–13.0)	7.0 (3.0–12.5)	0.87
Median social support significant 0ther (IQR)	6.0 (4.4–6.8)	6.8 (5.3–7.0)	0.04	6.0 (5.0–7.0)	6.8 (5.8–7.0)	0.039	6.0 (5.1–6.9)	7.0 (5.5–7.0)	0.039
Median social support family (IQR)	5 (2.9–6.1)	6.3 (5.0–7.0)	0.012	5 (3.3–6.5)	6.3 (5.5–7.0)	< 0.001	5.5 (4.6–6.5)	6.3 (5.1–7.0)	0.072
Median social support friends (IQR)	5.3 (3.9–6.1)	5.8 (5.0–6.5)	0.16	5.3 (4.1–5.9)	5.8 (5–6.5)	0.03	5.3 (4.6–6.4)	5.8 (5–6.4)	0.305
Median social support total (IQR)	5.5 (3.6–6.2)	6.1 (5.3–6.6)	0.03	5.3 (4.0–6.2)	6.1 (5.4–6.7)	0.006	5.6 (4.5–6.3)	6.1 (5.4–6.7)	0.116

*Note:* ‘Cannot work at all’ are those who did not return to work following injury. ’Decreased hours’ are those who returned to work but worked less hours in a week following injury. ‘Decreased productivity’ are those who returned to work for the same number of hours per week but reported achieving less at work compared to before the injury. Mean scores were calculated for each subgroup (significant others, family, friends). A higher mean score indicates higher levels of perceived support. All analyses were Mann Whitney U tests (2 sided).

There was no significant difference in sex or ethnicity between the varying degrees of work following injury. Vehicle‐ and sporting‐related injuries were associated with not being able to work at all following injury. No individual mechanism was significantly associated with RTW with decreased hours or decreased productivity. An ISS score of over 16 was associated with an inability to RTW and an ability to RTW but with decreased hours. Injuries requiring admission to ICU were also significantly associated with an inability to RTW and an ability to RTW but with decreased hours. There was no difference in work status and admitting specialty. Having a GCS of less than 15 on arrival to ED was associated with an inability to RTW, but there was no difference in the decreased hours and decreased productivity groups.

Participants with probable anxiety at follow up were more likely to have all three degrees of decreased work following injury. Those with probable depression at follow up were more likely to be unable to RTW at all or only RTW with decreased hours, but not with decreased productivity. When the three mental illnesses were combined, this combined variable was associated with all three degrees of decreased work. In terms of pre‐injury mental illness, anxiety and depression were associated with not being able to work at all but not decreased hours or productivity.

A longer hospital LOS was associated with a complete inability to RTW. Lower social support from significant others was associated with decreased hours, decreased productivity and an inability to RTW at all following injury.

Results of the multivariable analysis are displayed in Table 5. All individuals in this analysis were working full time prior to injury. An older age, longer LOS and probable depression were all significantly associated with decreased work following injury. The association between depression and decreased work was strong—with an odds ratio of 5.29. There was no evidence of multicollinearity in the final step of the model as indicated by the variance inflation factors.

**TABLE 5:** Multivariable association between explanatory variables and decreased work following injury.

Variable	Step 1 odds ratio (95% confidence interval)	Variance inflation factor (Step 1)	Final step odds ratio (95% confidence interval)	Variance inflation factor (final step)	*p* (final step)
Increasing age	1.10 (1.04–1.16)	1.19	1.08 (1.04–1.13)	1.06	< 0.01
Male Sex	0.56 (0.16–2.01)	1.26	0.36 (0.14–1.03)	1.05	0.058
Longer hospital LOS	1.07 (1.01–1.13)	1.93	1.04 (1.00–1.09)	1.07	0.048
ICU admission	2.89 (0.77–10.81)	1.72			
Probable PTSD at follow up	0.29 (0.05–1.58)	1.70			
Probable anxiety at follow up	124.22 (4.52–undefined)	7.08			
Probable Depression at follow up	12.93 (1.82–91.99)	2.65	5.29 (1.90–14.69)	1.10	0.01
Probable PTSD, anxiety or depression at follow up	0.02 (0–0.47)	9.28			
GCS 15 in ED	4.48 (0.67–29.89)	1.43			
Social support—significant other	1.23 (0.8–1.91)	2.08			
Social support—family	0.61 (0.38–0.99)	2.68			
Social support—friends	1.46 (0.89–2.39)	2.17			
ISS score 16+	0.6 (0.21–1.71)	1.30			
Pre‐injury PTSD	Uncalculatable	1.55			
Pre‐injury anxiety	59.8 (0.55–Undefined)	13.67			
Pre‐injury depression	18.17 (0.77–430.93)	2.66			
Pre‐injury PTSD, depression, or anxiety diagnosis	0.03 (0–3.31)	16.24			
Constant	Uncalculatable		0.0013		

*Note:* ‘Social support’ was determined by the MSPSS questionnaire, where participants ranked their perceived level of support on a seven‐point Likert scale. Mean scores were calculated for each subgroup (significant others, family, friends). A higher mean score indicates higher levels of perceived support. ‘Probable PTSD’ refers to those who were likely to have PTSD, based on a pre‐determined threshold from the PCL‐5 questionnaire. ‘Probable anxiety’ refers to those who were likely to have anxiety, based on a pre‐determined threshold from the HADS questionnaire. ‘Probable depression’ refers to those who were likely to have depression, based on a pre‐determined threshold from the HADS questionnaire.

## 4 Discussion

This study provides valuable insights into the factors influencing RTW following major trauma in New Zealand. Consistent with previous literature, physical factors such as age, longer hospital LOS, and ICU admission were significantly associated with a reduced ability to return to full‐time work [15, 16]. This study adds to the limited body of evidence regarding the psychological impacts of trauma on occupational outcomes by demonstrating strong associations between post‐injury depression and reduced RTW. However, depression may be both a cause and correlate of not RTW, necessitating further interventional research to elucidate the direction of this relationship.

Probable PTSD did not emerge as being significantly associated with reduced RTW. This may be due to the relatively small number of participants meeting PTSD diagnostic thresholds, thus decreasing power. There were more people above threshold for anxiety and depression, although the effect size was larger for these too. Ultimately, probable depression was strongly associated with decreased work in the final step of our multivariable analysis; however, this was confounded with other mental illnesses (including PTSD) in Step 1. It should be noted that PTSD was not significant in univariable or multivariable analyses.

Interestingly, work‐place related injuries were significantly less likely to result in reduced RTW, which may reflect the benefit of institutional rehabilitation support linked to occupational injury claims. This observation may be attributed to the advantages provided by ACC, which offers income support to injured workers, enabling them to RTW in a reduced capacity until they are able to resume their duties in full [13]. However, the numbers in this subset analysis were small, so a larger sample size is required to confirm this observation.

This study was not powered to draw definitive conclusions about ethnic disparities, and the low representation of Māori participants limits meaningful subgroup analysis. Nonetheless, this observation reinforces ongoing concerns about inequities in research participation and healthcare access for Māori. In Aotearoa New Zealand, Māori experience a disproportionate incidence of major trauma, associated with a higher burden of morbidity and mortality [24]. Furthermore, Māori experience inequities with accessing rehabilitation services, which can delay recovery following traumatic injuries [25]. As such, further investigation into identifying specific barriers to rehabilitation for Māori is required to ensure equitable outcomes in post trauma care in Aotearoa.

These findings reinforce the importance of comprehensive post‐trauma care that addresses both physical and mental health components to optimise recovery, and highlights the critical impact of depression on functional outcomes following trauma. These findings align with previous literature which suggests that psychological sequelae following trauma are common and potent predictors of long‐term disability [4, 5, 6, 7]. Structured follow‐up pathways that incorporate psychological assessment, referral and support services may mitigate the long‐term occupational impairments associated with untreated mental illness following trauma.

In Aotearoa New Zealand, ACC provides compensation and treatment for both physical and mental injury—such as PTSD as a sequelae of a traumatic injury [13]. A related study has reported that those meeting the threshold for PTSD were more likely to report ACC‐related problems than those below the threshold [26]. As such, standardised ACC referral for psychological support in traumatically injured patients could improve recovery from the psychological sequelae of trauma. Given this study's finding of mental injury being significantly associated with reduced RTW, improving access to ACC funding for psychological injury could be a possible solution to improving earlier RTW. Most people with probable PTSD are undiagnosed [27]. As stated by Ryznar and Balogh, ‘Psychological screening should be incorporated into routine trauma predischarge and follow‐up checklist rather than the optional or ‘when suspected’ approach’ [28].

That being said, this was observational research and different causal pathways cannot be ruled out. For example, injury severity (either measured directly or via proxy such as LOS) could cause the depression which causes the lower RTW. Alternatively, failing to return to work could impact self‐esteem and economic wellbeing and henceforth lead to depression. A final possibility is that the injury caused the depression and lower RTW independently and not through a mediation pathway. Interventional research would better answer the question regarding which pathways are more likely, although it is likely improving psychological outcomes does improve RTW as per previous research [29, 30, 31].

### 4.1 Limitations

There are several limitations to this study. The modest sample size may have impacted the statistical power of some analyses. The low response rate of 24.3% raises the potential for responder bias, limiting the generalisability of these findings. Notably, younger male, non‐Caucasian participants were less likely to respond. The delays between the injury and study invitation and completion may have lowered the response rate. In future, a more prospective approach regarding the psychological aspects of the study with engagement at the time of initial hospitalisation and utilisation of other methods such as follow‐up at outpatient clinics afterwards should lead to a higher response rate. Despite this, valuable analyses were performed that identified significant predictors of RTW.

The mixed retrospective plus cross‐sectional study design and self‐reported outcomes introduce the potential for recall bias for questions which required the participant to recall information from their past regarding their employment or prior mental health. Participants were recruited between May 2016 and March 2022, but all participants completed questionnaires between March and July 2022. As such, recall bias had a greater impact on those who were injured earlier in the recruitment period. Thresholds for PTSD, anxiety and depression were obtained by self‐reported questionnaires and ideally these should be diagnosed via clinical interview. Furthermore, while multivariable analyses were performed, residual confounding from unmeasured factors—such as job type, employer flexibility, or social determinants of health—cannot be excluded.

In terms of Māori, there was a lower response rate and sample size. In addition, the questionnaires were not validated within a Te Ao Māori worldview. Most relevantly, the MSPSS did not include culturally specific language for Māori (e.g., use of words such as whānau and iwi). There is a need for more specific targeting of Māori appropriately in future research although it should be noted a Māori consultation was undertaken as per the requirements for HDEC.

All responses were received after the start of the Covid‐19 pandemic, although no injuries were reported after Covid‐19. This introduces an obvious potential confounder for many people. The design of the questionnaires was undertaken before the pandemic. Nevertheless, many people did return to work after injury and meaningful analysis was still able to be undertaken.

## 5 Conclusions

This study highlights the physical and psychological factors that influence RTW following major trauma, with probable depression emerging as the strongest predictor of reduced occupational functioning. These findings underscore the need for integrated trauma care that includes early screening and intervention for mental health conditions in traumatically injured patients. To improve long‐term outcomes, trauma rehabilitation should adopt a holistic approach that addresses both the physical and psychological needs of patients.

## Funding

This work was supported by a University of Otago Research Grant, UORG #3343.

## Ethics Statement

Ethical approval was provided by the New Zealand Health and Disability Ethics Committee (Reference Number 20/CEN/8/AM03).

## Conflicts of Interest

Laura Joyce is a section editor for EMA. The authors declare no conflicts of interest.

## Supporting information


**Table S1:** Demographics of responders vs. non‐responders.

**Figure S1:** Flowchart of study participants.

**Data S1:** Supporting Information.

## Data Availability

The data that support the findings of this study are available from the corresponding author upon reasonable request.
