# Supplementary material for: Predictors of Return to Work Following Major Trauma in New Zealand
Source: Emerg Med Australas. 2026 Oct 29;38(6):e70382. doi: 10.1111/1742-6723.70382 (PMC13717470; doi:10.1111/1742-6723.70382)
Supplement: Supplementary file 1 [file EMM-38-0-s001.docx]

# **Supplementary Material 1: Work Questionnaire**

1. Did you work before your injury alluded to for this study? *(tick one or more)*

- Yes, full time
- Yes, part time
- No, not at all
- Other (e.g. volunteered, was a full time student, pensioner, etc.)___________________

2. Are you working now?

- Yes, full time
- Yes, part time
- No, not at all
- Other (e.g., full time student) ______________________

3. Did you return to work in any capacity after your injury?

- Yes
- No

4. If you have returned to work in any capacity, please complete the following two statements (*leave this blank if you have not returned to work*):

- I returned to light duties (<80% of what you would normally do) after _______________ weeks/months/years
- I returned to the same duties (≥80% of what you would normally do) I had before the injury after _____ weeks/months/years (if you have not yet returned or will not return to the same duties you had before the injury, write “never” into the blank space)

5. Overall, as at the present time, are you experiencing a decline in work capacity as a direct result of the injury sustained? *(tick one or many)*

- Yes, cannot work at all
- Yes, decreased hours
- Yes, productivity (amount of work you can do per hour)
- No decrease in work capacity (skip next question)

6. What is the MAIN reason you have experienced a decline in work capacity as a direct result of the injury sustained? *(skip this question if you have not experienced this)*

- Cognitive impairment including loss of ability to concentrate for long enough periods of time
- Psychological distress (e.g., depression, anxiety, post-traumatic stress disorder)
- Pain
- Loss of sleep
- Loss of mobility
- Other (please specify) _____
