# Supplementary material for: Predictors of Return to Work Following Major Trauma in New Zealand
Source: Emerg Med Australas. 2026 Oct 29;38(6):e70382. doi: 10.1111/1742-6723.70382 (PMC13717470; doi:10.1111/1742-6723.70382)
Supplement: Supplementary file 3 [file EMM-38-0-s003.docx]

Supplementary Table 1: Demographics of responders vs. non-responders

| **Variable** | **Responders (n=203) n (%) or mean (standard deviation) or median (interquartile range)** | **Non-responders (n=633) n (%) or mean (standard deviation) or median (interquartile range)** | **P-value^a^** |
| --- | --- | --- | --- |
| Mean age | 54.4 (15.6) | 44.4 (18.9) | **<0.001** |
| Male sex | 139 (69%) | 486 (77%) | **0.023** |
| Median ISS | 16 (13-20) | 17 (14-22) | **0.029** |
| Mean years between injury & initial study invitation | 2.8 (0.7) | 2.8 (0.7) | 0.170 |
| Alcohol use (on admission) | 10 (45%) | 47 (7%) | 0.285 |
| Mean hospital LOS (days) | 11.0 (2.5) | 10.4 (11.9) | 0.588 |
| ICU admission | 47 (23%) | 152 (24%) | 0.833 |
| *Ethnicity* | | | |
| European | 191 (94%) | 509 (80%) | **<0.001** |
| Māori | 7 (3%) | 64 (10%) |  |
| Asian | 1 (1%) | 26 (4%) |  |
| MELAA^b^ | 1 (1%) | 4 (1%) |  |
| Pacific Peoples | 0 (0%) | 16 (3%) |  |
| All others | 3 (2%) | 14 (2%) |  |
| *Mechanism of injury^c^* | | | |
| Vehicle-related | 106 (52%) | 345 (55%) | 0.626 |
| Sports-related | 61 (30%) | 102 (16%) | **<0.001** |
| Fall | 29 (14%) | 130 (21%) | 0.061 |
| Workplace-related | 8 (4%) | 31 (5%) | 0.711 |
| Assault | 1 (1%) | 40 (6%) | **0.002** |
| Unknown | 1 (1%) | 10 (2%) | 0.421 |

^a^ Pearson Chi-Square test (2 sided) with continuity correction used for 2x2 cross tabulated count data except for Unknown mechanism which used doubled Fisher 1 tailed; Ethnicity compared using Fisher-Freeman-Halton Exact Test (2 sided); t-test for independent means (2 sided) used for all continuous variables except ISS which was compared using Mann-Whitney U test.

^b^ MELAA: Middle Eastern, Latin American, and African
^c^ A patient can have more than one mechanism of injury.
