# Supplementary material for: Predictors of Return to Work Following Major Trauma in New Zealand
Source: Emerg Med Australas. 2026 Oct 29;38(6):e70382. doi: 10.1111/1742-6723.70382 (PMC13717470; doi:10.1111/1742-6723.70382)
Supplement: Supplementary file 4 [file EMM-38-0-s002.docx]

**Supplementary Figure 1.** **Flowchart of study participants.**
